# Evaluating cone-beam computed tomography vs. intraoral radiography for the detection of endodontal disease in dogs: diagnostic performance and inter-rater agreement

**DOI:** 10.3389/fvets.2026.1893094

**Published:** 2026-08-05

**Authors:** Serena Bonacini, Stephanie Goldschmidt, Boaz Arzi, Natalia Vapniarsky, Milinda Lommer, Andrew Blandino, David Hatcher, Maria Soltero-Rivera

**Affiliations:** 1 William R. Pritchard Veterinary Medical Teaching Hospital, School of Veterinary Medicine, University of California, Davis, Davis, CA, United States; 2 Department of Surgical and Radiological Sciences, School of Veterinary Medicine, University of California, Davis, Davis, CA, United States; 3 Department of Pathology, Microbiology and Immunology, School of Veterinary Medicine, University of California, Davis, Davis, CA, United States; 4 Aggie Animal Dental Center, Mill Valley, CA, United States; 5 Department of Statistics, University of California, Davis, Davis, CA, United States

**Keywords:** apical periodontitis, canine dentistry, cone beam computed tomography, dental radiography, endodontal disease, oral medicine

## Abstract

**Introduction:**

The comparative diagnostic utility of cone-beam computed tomography (CBCT) and intraoral dental radiography (IDR) for endodontal disease in dogs remains unclear. Accordingly, this study evaluated both imaging modalities against histopathologic findings as the gold standard.

**Methods:**

In this prospective observational study, client-owned dogs undergoing evaluation for suspected endodontal disease were imaged with both CBCT and IDR under general anesthesia. Forty-six teeth from 16 dogs were included. Four radiographic features—periapical lucency, failure of pulp canal narrowing, external inflammatory root resorption, and loss of crown integrity—were independently scored by five blinded evaluators using a standardized qualitative scale. Histopathological assessment of pulp vitality was available for 18 teeth. Diagnostic performance, inter-rater agreement, and modality-related effects were analyzed using mixed-effects models and concordance statistics.

**Results:**

CBCT was associated with significantly greater detection of imaging features associated with endodontal disease than IDR overall (*p* < 0.002), particularly for periapical lucency (*p* < 0.001). Both modalities demonstrated high sensitivity for identifying non-vital teeth, but limited specificity, reflecting disease prevalence within the histopathology subset. Inter-rater agreement was modest for both modalities and was affected primarily by scoring variability among evaluators rather than systematic differences in scoring patterns.

**Discussion:**

CBCT demonstrated superior detection of periapical lucency compared with IDR in dogs. Nonetheless, inter-rater variability remains a significant limitation, emphasizing the need for standardized image interpretation and integration with clinical findings.

## Introduction

Endodontal disease is a common condition in dogs, with a reported prevalence of at least 27% (1). Endodontal disease begins with an insult that results in inflammation of the dental pulp (pulpitis), which then results in reversible or irreversible pulpitis, pending the severity of the insult. Irreversible pulpitis is defined as pulpitis that results in pulp necrosis. This process is driven by bacterial invasion of the root canal system and the release of toxic by-products within the devitalized tooth (2, 3). Once the infection or its by-products extends beyond the apical delta the interaction between microbial pathogens and the host immune response commonly results in periapical tissue damage, clinically recognized as apical periodontitis (2, 3). The early stages of these pathological changes are often subtle and challenging to detect using conventional diagnostic modalities.

When not diagnosed and treated in a timely manner, endodontal disease will result in chronic pain and functional impairment (1, 4, 5). Early and accurate recognition of endodontal disease is therefore essential to minimize chronic pain, enable tooth-saving treatment options, and optimize clinical outcomes in dogs (1, 4, 5).

In veterinary medicine, intraoral dental radiography (IDR) remains the most commonly used imaging technique for assessing endodontal disease (6, 7). Historically, radiographic diagnosis of endodontal disease has been based on the identification of specific radiographic features, including periapical lucency, failure of normal root canal narrowing, external inflammatory root resorption (EIRR), and loss of crown integrity (e.g., crown fractures, crown-root fractures, or severe attrition/abrasion with pulp exposure) (8). However, histopathological examination—while considered the gold standard for detecting apical pathology—is destructive and unsuitable for clinical diagnosis, especially when tooth preservation is desired (2, 7).

IDR is limited by its two-dimensional nature and the subsequent geometric distortion, superimposition of anatomical structures and operator-dependent variables associated with this imaging modality (7). Several studies have demonstrated the limited sensitivity of IDR for detecting early periapical disease. In veterinary patients, up to 57.7% of histologically confirmed apical periodontitis cases may show no radiographic abnormalities (4, 7, 9, 10). Comparable findings are reported in human dentistry, where conventional radiography fails to identify approximately 25% of apical lesions (11). These limitations underscore the need for advanced imaging modalities capable of reliably detecting early pathological changes.

Cone-beam computed tomography (CBCT) has been established in human dentistry as a highly sensitive imaging modality for the detection of endodontal and periapical disease (12, 13). By enabling three-dimensional visualization of dental and maxillofacial structures, CBCT has demonstrated superior diagnostic accuracy compared with both IDR and conventional computed tomography (CT). Human studies indicate that CBCT allows earlier detection of periapical lesions than IDR, particularly during the initial stages of lesion development (14, 15).

The clinical application and diagnostic performance of CBCT for endodontal disease in veterinary patients remain insufficiently investigated. We hypothesize that CBCT provides improved visualization and detection of radiographic signs of endodontal disease compared with conventional IDR in dogs. The aim of this prospective study is to compare CBCT and IDR in terms of diagnostic performance, using histopathological examination of the dental pulp as the reference standard, when available, and to evaluate the potential clinical implications of CBCT-based diagnosis.

## Materials and methods

The current study was designed as a prospective observational study conducted at the Dentistry and Oral Surgery Service (DOSS), “William R. Pritchard” Veterinary Medical Teaching Hospital (VMTH), School of Veterinary Medicine (SVM), University of California, Davis (UCD). The study was approved by the Institutional Animal Care and Use Committee (IACUC; protocol no. 23672).

Client-owned dogs were prospectively enrolled in the study from November 2023 to August 2024. Written informed consent was obtained from all owners prior to enrollment. Dogs of any age, gender, neuter status, breed and body weight, who were presented to the UCD SVM VMTH DOSS for evaluation and treatment of endodontal disease under general anesthesia were considered adequate candidates for study enrollment. Due to minimally prolonged anesthetic time, dogs of ASA status III or above were not considered eligible for enrollment.

Demographic variables such as age, gender, neuter status, breed, body weight along with type of tooth affected by endodontal disease and type of treatment pursued—extraction vs. root canal treatment (RCT)—were recorded for each patient in the study. Cases were eligible if they had endodontal disease on clinical oral examination, defined as any of the following: complicated crown or crown-root fracture, root fracture, attrition/abrasion with pulp exposure, or intrinsic discoloration. Dogs with deciduous dentition, previous endodontal or restorative therapy, odontodysplasia or who were medically or intrinsically immunosuppressed were not considered eligible for enrollment in the present clinical trial.

Once under general anesthesia, each patient underwent both cone-beam computed tomography (CBCT) and intraoral dental radiography (IDR) of the affected tooth. CBCT was performed first, followed immediately by IDR.

A NewTom 5G CBCT unit (NewTom QR S.r.l., Verona, Italy) was used for image acquisition with a preset exposure time of 26 s, a field of view ranging from 15 × 15 cm to 15 × 22 cm based on patient head size, and a voxel resolution of 150 μm. CBCT images were reviewed using Anatomage In Vivo7 imaging software (Anatomage Inc., San Jose, CA, USA), which allowed image visualization in axial, sagittal, and coronal planes as well as three-dimensional reconstruction of the skull.

Intraoral dental radiographs were acquired using a conventional indirect dental radiography system (Midmark Preva DC Intraoral X-ray System). Exposure settings (kVp, mA, and ms) were adjusted according to patient size, whereas image resolution was consistently set at 55 μm. Radiographic images were evaluated using Metron Dental Imaging software (EponaTech LLC, Paso Robles, CA, USA).

**Figure 1:**
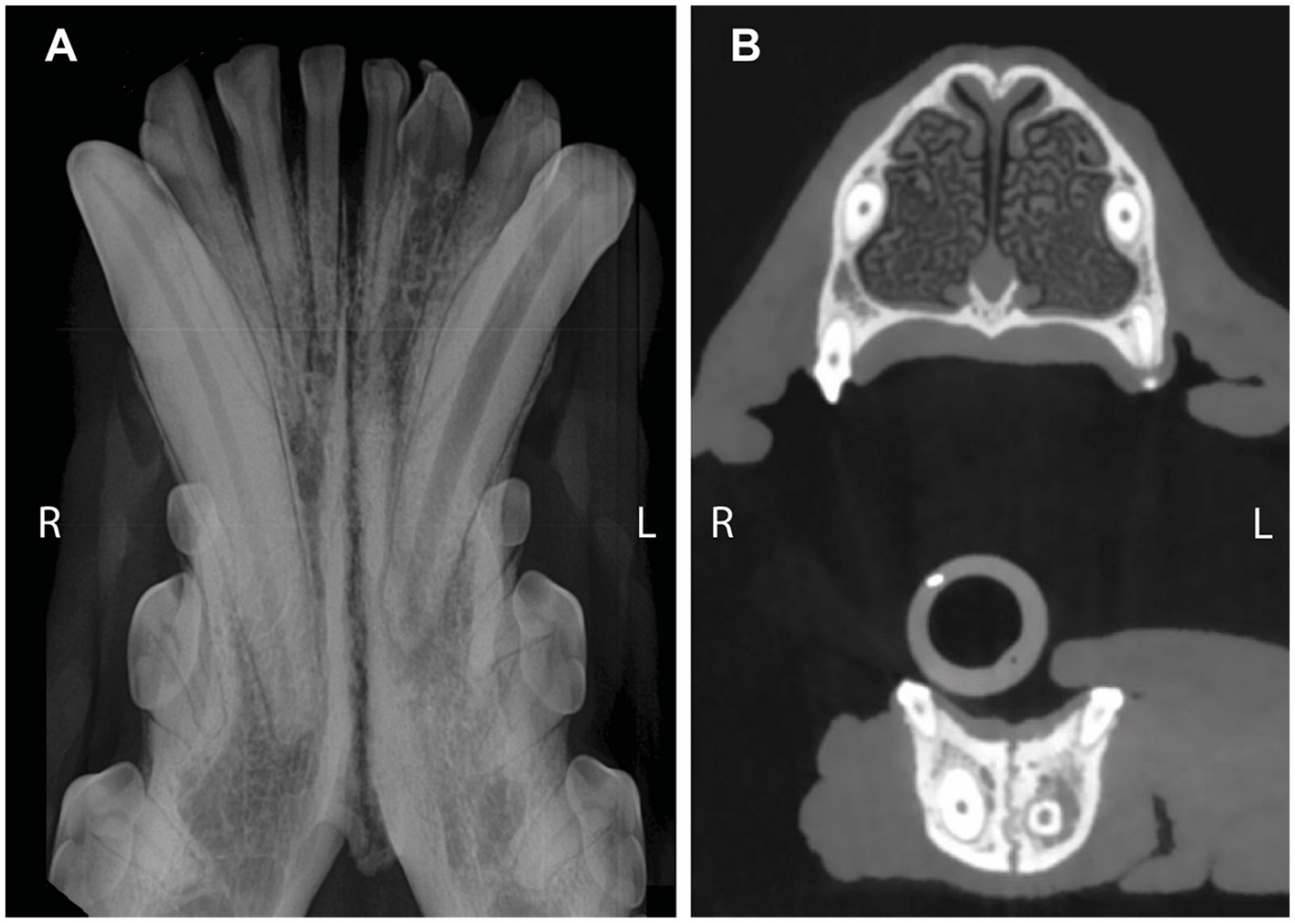
**(A)** Occlusal radiographic view of the mandibular canine and incisor teeth in a 3-year-old, female spayed, German Shepherd dog, presenting for complicated crown fracture of the left mandibular canine tooth. Radiographic findings include periapical lucency, failure to narrow of the root canal, external inflammatory root resorption (EIRR), and loss of crown integrity of the left mandibular canine tooth. Note additional findings, such as loss of crown integrity of the left mandibular third incisor tooth and failure to narrow of the root canal and replacement root resorption of the left mandibular second incisor tooth. **(B)** Cone-beam computed tomography (CBCT) axial view of the mandibular canine teeth of the same patient in 1A-−3 year-old, female spayed, German Sheperd dog, presenting for complicated crown fracture of the left mandibular canine tooth. Radiographic findings include periapical lucency, failure to narrow of the root canal, EIRR. Note the sharply demarcated and well-defined margins of the periapical lucency and the wider root canal of the left mandibular canine tooth, compared to the contralateral side.

**Figure 2:**
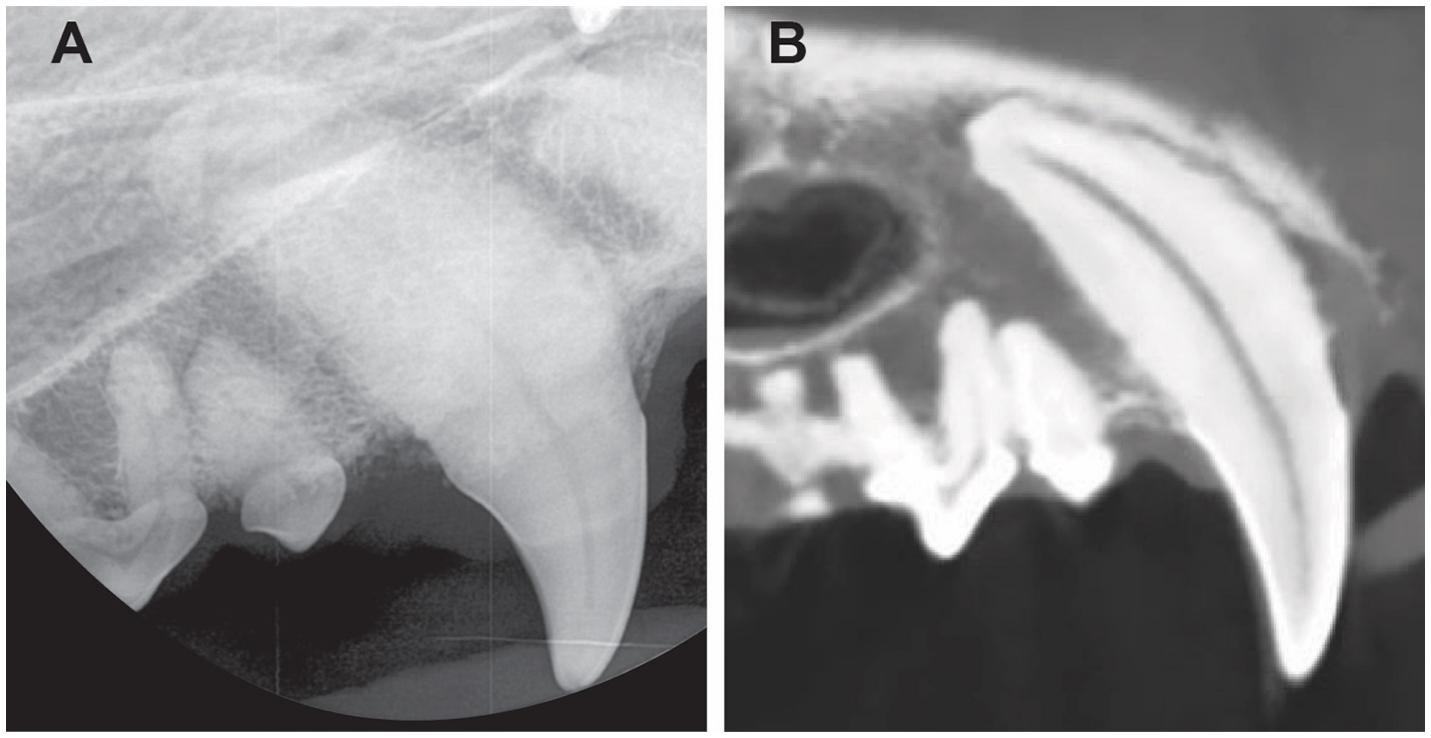
**(A)** Intraoral radiograph of the right maxillary canine tooth in a 10-year-old female spayed Labrador Retriever presented for intrinsic discoloration of her right maxillary canine tooth. **(B)** Cone-beam compued tomography (CBCT) sagittal view of the right maxillary canine tooth of the same patient in **(A)**. Note the replacement root resorption affecting the mesial aspect of the root structure of the right maxillary canine tooth, characterized by loss of normal periodontal ligament space and replacement of root tissue by surrounding alveolar bone. This process is distinct from EIRR evaluated elsewhere in this study.

Four main radiographic features of endodontal disease were considered for image evaluation for both CBCT and IDR: periapical lucency, root canal failure to narrow, external inflammatory root resorption (EIRR) and loss of crown integrity (8) (Figures 1, 2). Similar to methodology previously applied for other studies from our group (16, 17), a subjective qualitative scoring system for each radiographic feature was established as follows: 0 = inability to identify, 1 = poor visualization, 2 = fair visualization, 3 = excellent visualization.

The same scoring system was applied to each imaging modality for every tooth.

Both CBCT and IDR images obtained from the enrolled dogs were randomized. Five evaluators (SB, resident in Veterinary Dentistry and Oral Surgery; MSR, SG, BA, ML, board-certified veterinary dentists and oral surgeons) were recruited for image interpretation. Prior to image interpretation, a calibration meeting was held to standardize scoring criteria and ensure consistency among evaluators. Each evaluator was kept blinded from all the other evaluator scores. CBCT and IDR image sets were randomized independently, and evaluators were unaware of which intraoral radiographs corresponded to a given CBCT examination. Intraoral dental radiographs were evaluated first to prevent any potential bias in the interpretation of CBCT images.

In addition to qualitative image evaluation, a quantitative assessment of periapical lucencies was performed when lesions were identifiable on either imaging modality. The maximum linear dimension of each periapical lucency was recorded, defined as the greatest measurable width of the lesion across any plane for CBCT or within the two-dimensional projection for IDR, as reported in previous studies (18). In multirooted teeth with multiple associated periapical lesions, measurements were obtained for each root, and the mean value was calculated to yield a single representative measurement per tooth. Teeth were subsequently categorized according to tooth type for downstream analysis.

Measurements of periapical lucency (PAL) dimensions were collected for descriptive purposes only. Statistical comparisons of PAL size between CBCT and IDR were not performed because PAL measurements were not available for equivalent lesion populations across the two imaging modalities. Because the primary objective of the study was to evaluate lesion detection rather than lesion size characterization, PAL measurements were retained as descriptive data only.

After imaging acquisition, any required dental treatment (extraction vs. RCT) was pursued as per agreement with the owner. For 18 teeth, the extracted teeth or pulp removed while performing RCT were put into 10% buffered formalin and submitted for histopathological confirmation of endodontal disease and tooth vitality or non-vitality.

**Figure 3:**
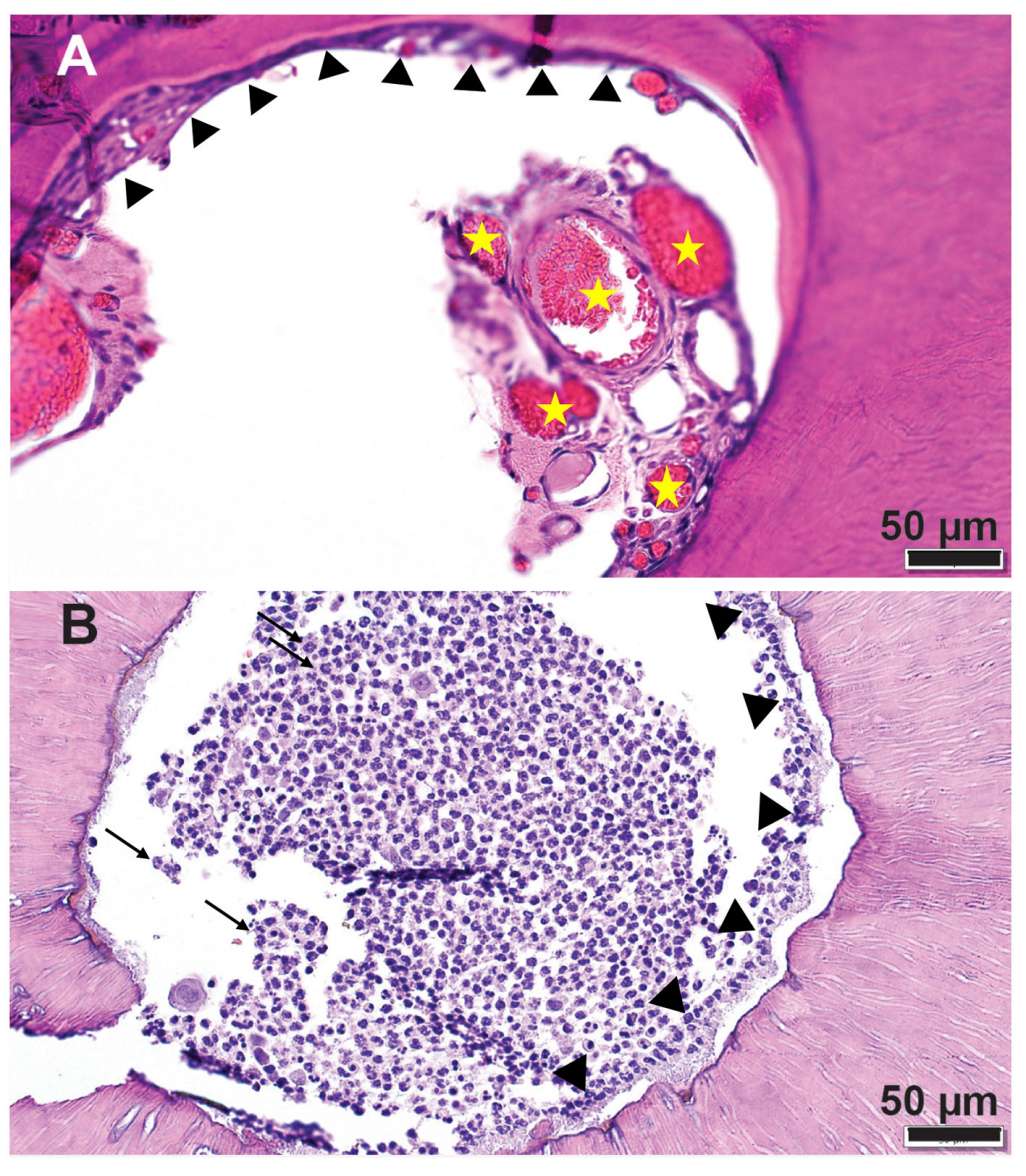
**(A)** Hematoxylin and eosin (H&E) stained histological section (20x magnification) of a transversely-sectioned tooth in a 3-year-old, female spayed, German Sheperd dog, presenting for a complicated crown fracture. The dental pulp demonstrates a well-defined odontoblastic layer (black arrowheads) adjacent to the dentinal wall, with visible pulp vasculature (yellow stars), indicating a viable pulp. With these findings, the tooth is considered vital. **(B)** Hematoxylin and eosin (H&E) stained histological section (20 × magnification) of a transversely-sectioned tooth in a 3-year-old, female spayed, Pug, presenting for a complicated crown fracture of the right maxillary fourth premolar. The dental pulp cavity is filled with variable degenerate polymorphonuclear cells (neutrophils) (black arrows) and segmental remnants of viable odontoblasts along the lower right inner dentinal wall, supporting a diagnosis of inflammatory pulp changes (pulpitis) in a vital tooth.

**Figure 4:**
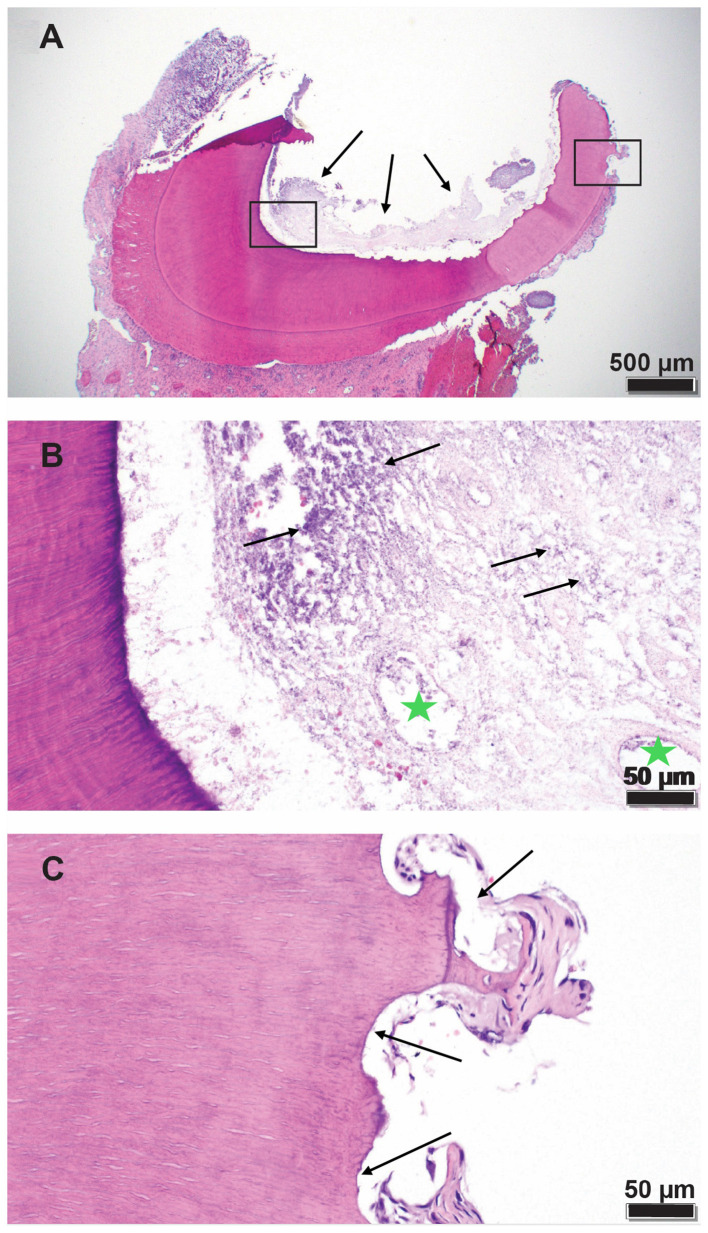
**(A)** Low-magnification (2 × ) hematoxylin eosin (H&E) stained histological section of a transversely-sectioned right maxillary canine tooth with a complicated crown fracture in a 1-year-old, male intact, Bernese Mountain dog. The pulp tissue is replaced by an amorphous amphophilic granular material (black arrows), and no evidence of odontoblasts or vasculature, consistent with non-vital pulp. **(B)** Higher-magnification (20x) view of the the area enclosed in the black rectangle in **(A)**. Note the absence of viable odontoblasts and amorphous granular amhophilic material (suspect bacteria and mineralized necrotic tissue) (black arrows) with ghost profiles of blood vessels (green stars). **(C)** Higher-magnification (20x) detail of the area enclosed in the outer black rectangle in **(A)**. Note multiple resorption lacunae of the cementum layer (black arrows).

The teeth were decalcified in 15% formic acid prior to trimming and paraffin embedding and stained with hematoxylin and eosin as previously reported by our group (19). Specimens were classified as vital or non-vital based on whether viable pulp was observed on microscopic evaluation performed by a board-certified veterinary anatomic pathologist (NVA) (Figure 3). Teeth showing mixed pulpal status, with necrosis in one root and vitality in others, were classified as non-vital, as pulpal necrosis in a single root was considered indicative of a progressive process ultimately affecting the entire tooth vitality (Figure 4).

### Statistical analysis

Diagnostic accuracy for CBCT and IDR was assessed using histopathology as the reference standard for pulp vitality only in the subset of teeth with available histologic results (*n* = 18); all other statistical analyses were conducted across the entire study population. Diagnostic performance was quantified using sensitivity, specificity, positive and negative predictive values, overall accuracy, Cohen's kappa coefficient, balanced accuracy when appropriate, and McNemar's test for paired binary outcomes. Because five raters contributed ordinal diagnostic scores, two approaches were used to generate binary diagnostic outcomes: (1) application of a decision rule to each rater's score followed by aggregation of the resulting classifications (pre-average), and (2) averaging of ordinal scores across raters prior to applying the binary decision rule (pre-median, post-median). Confusion matrices were constructed for each imaging modality under both approaches to provide a consistent structure for comparison.

Inter-rater reliability, rater bias, and rater precision were evaluated for each diagnostic modality (CBCT and IDR) and were assessed with Concordance correlation coefficients and their decomposition into accuracy (bias) and precision (correlation) coefficients. A linear mixed effect model was constructed to explain systematic differences in scores. In this model, rater and patient identification number were specified as random effects, whereas diagnostic modality and diagnostic criterion (periapical lucency, root canal failure to narrow, external inflammatory resorption, and loss of crown integrity) were treated as fixed effects. Interaction terms involving rater, patient, diagnosis, and modality were included to determine whether agreement varied across diagnostic categories or between imaging methods. Significant interactions were interpreted as evidence of rater-dependent divergence or patient-specific variability in scoring.

All statistical analyses were performed using R (version 4.5.0) with statistical significance defined as *p* < 0.05 and values between 0.05 and 0.10 interpreted as trends.

## Results

### Study population, included teeth, and histopathology

Twenty-one client-owned dogs were initially enrolled between November 2023 and August 2024; 16 met the final inclusion criteria. Four dogs were excluded due to non-diagnostic radiographs, and one due to inclusion of a deciduous tooth.

A total of 46 teeth were included, all evaluated with both CBCT and IDR. Of these, 32 teeth were extracted, 9 underwent RCT, and 5 were not treated and subsequently classified as uncomplicated crown fractures following examination under anesthesia (Table 1).

**Table 1:** Patients demographics and variables, including breed, sex, age in years (ys), body weight in kilograms (kg), affected tooth type, type of injury, elected treatment option (extraction vs. root canal therapy), collected biopsy, tooth vitality vs. non-vitaly.

Patient	Breed	Sex	Age (ys)	Weight (kg)	Tooth	Injury	Extraction	Root canal therapy	Biopsy	Vital	Non-vital
1	Australian Cattle dog	Mc	7	23	RmaxP4	UCF	Yes	No	Yes	No	Yes
					LmaxP4	UCF	Yes	No	Yes	No	Yes
2	Mixed breed	Mc	5	34.6	LmaxC	Disc NV	No	Yes	No	N/A	N/A
					RmaxP4	UCF	No	No	N/A	N/A	N/A
					LmaxP4	CCF	No	Yes	No	N/A	N/A
3	German Sheperd	Fs	3	33.4	LmandC	CCF	Yes	No	Yes	Yes	No
					LmandI3	UCF	Yes	No	Yes	No	Yes
					LmandI2	UCF	Yes	No	Yes	No	Yes
					LmandI1	UCRF	Yes	No	Yes	No	Yes
4	Cane Corso	M	0.75	41	RmaxM2	Disc NV	Yes	No	Yes	No	Yes
					Supernumerary RmaxI2	UCRF	Yes	No	No	N/A	N/A
5	Dog De Bordeaux	Fs	6	60	LmaxC	AT	No	No	N/A	N/A	N/A
6	Mixed breed	Fs	3	41	LmandI3	CCF	No	Yes	Yes	Yes	No
7	Pug	Fs	3	6.5	RmaxP4	CCRF	Yes	No	Yes	Yes	No
					RmaxM1	Disc NV	Yes	No	Yes	No	Yes
8	Labrador Retriever	Fs	10	35.8	RmaxC	Disc NV	Yes	No	Yes	No	Yes
					LmaxC	Disc NV	Yes	No	Yes	No	Yes
9	German Sheperd	Mc	6	40.5	LmaxP4	CCRF	Yes	No	No	N/A	N/A
					LmaxC	AB PE	No	Yes	No	N/A	N/A
					RmaxC	AB PE	No	Yes	No	N/A	N/A
					LmaxP2	AB PE	Yes	No	No	N/A	N/A
					LmaxP3	AB PE	Yes	No	No	N/A	N/A
					RmaxP1	AB PE	Yes	No	No	N/A	N/A
					RmaxP2	AB PE	Yes	No	No	N/A	N/A
					RmaxP3	AB PE	Yes	No	No	N/A	N/A
					RmandP2	AB PE	Yes	No	No	N/A	N/A
					RmandP3	AB PE	Yes	No	No	N/A	N/A
					LmandP2	AB PE	Yes	No	No	N/A	N/A
					LmandP3	AB PE	Yes	No	No	N/A	N/A
10	Japanese Chin	Fs	4	5.1	RmaxC	NV	No	Yes	No	N/A	N/A
					LmaxC	NV	No	Yes	No	N/A	N/A
11	Pitt Bull Terrier	Fs	4	19.3	LmandC	CCF	No	Yes	No	N/A	N/A
12	Mixed breed	Fs	6	39.8	RmaxC	CCF	No	Yes	No	N/A	N/A
					LmaxI2	CCRF	Yes	No	Yes	No	Yes
					RmaxP4	UCF	No	No	N/A	N/A	N/A
13	Welsh Corgi	Mc	3	12.5	RmaxP4	UCF	No	No	N/A	N/A	N/A
					LmaxP4	UCF	No	No	N/A	N/A	N/A
14	Labradoodle	Fs	6	33.9	LmaxP4	CCRF	Yes	No	Yes	No	Yes
15	Miniature Schnauzer	Fs	1	3.2	RmandI2	Disc NV	Yes	No	Yes	No	Yes
					LmandI2	Disc NV	Yes	No	Yes	No	Yes
					LmandI3	Disc NV	Yes	No	Yes	No	Yes
					RmaxP4	Disc NV	Yes	No	Yes	No	Yes
16	Australian Cattle dog	Fs	2	13.7	LmandI2	RF	Yes	No	No	N/A	N/A
					LmandI3	RF	Yes	No	No	N/A	N/A
					RmaxM1	CCRF	Yes	No	No	N/A	N/A
					RmaxM2	CCRF	Yes	No	No	N/A	N/A

Mc, male castrated; M, male intact; Fs, female spayed; UCF, uncomplicated crown fracture; CCF, complicated crown fracture; UCRF, uncomplicated crown-root fracture; CCRF, complicated crown-root fracture; RF, root fracture; Disc NV, discoloration, non-vital tooth; NV, non-vital tooth; AB PE, abrasion with pulp exposure; AT, attrition. The tooth type has been reported according to the *Nomina Anatomica Veterinaria*.

A substantial number of periapical lesions (PAL) identified on CBCT were either not detectable or not sufficiently well-defined on IDR to permit reliable measurement. Restricting analysis to lesions measurable on both modalities would therefore have excluded many CBCT-detected lesions and introduced selection bias toward larger and more advanced lesions. Descriptive measurements of PAL for CBCT and IDR are summarized in (Table 2).

**Table 2:** Distribution of tooth types evaluated in the present study, including the number of teeth analyzed per tooth type and the corresponding periapical lucency (PAL) measurements (mm).

Tooth type	CBCT		IDR	
	Number of teeth	PAL size in mm	Number of teeth	PAL size in mm
MaxI2	1	4.53	1	4.6
MaxC	2	5.64	3	7.36
MaxP2	2	3.33	1	4.9
MaxP3	2	3.88	1	4.3
MaxP4	11	5.21	7	9.1
MaxM1	2	3.40	1	2.5
MaxM2	2	9.07	1	7.4
MandI1	1	3.36	-	-
MandI2	4	2.39	1	4.45
MandI3	4	2.45	1	11
MandC	2	5.64	2	7.75
MandP2	-	–	1	5
MandP3	2	4.83	–	–

Histopathology was available for 18 teeth (17 extracted and 1 treated with RCT). Among these, 15 teeth were classified as non-vital and 3 as vital (Table 3). Among the three teeth classified as vital, two exhibited histopathologic evidence of pulpitis, whereas the third showed limited evidence of pulpal vitality, with vascular endothelial structures present but no clearly viable odontoblastic layer. Histopathological evaluation was not available for the remaining teeth because adequate diagnostic pulp tissue could not be obtained, including due to pulp desiccation, extensive pulp damage during extraction, sectioning artifacts, or inadvertent disposal of the extracted tooth before histopathologic submission.

**Table 3:** Histopathologic findings and imaging-based diagnoses for all 18 tooth samples included in the study.

Patient	Tooth	Tooth vitality on histopathology	CBCT diagnosis				IDR diagnosis			
			PAL	FTN	EIRR	LCI	PAL	FTN	EIRR	LCI
1	RmaxP4	Non-vital	X		X	X	X	X	X	
	LmaxP4	Non-vital	X		X	X	X		X	X
3	LmandC	Vital	X	X	X	X	X	X	X	X
	LmandI3	Non-vital	X	X		X	X	X	X	X
	LmandI2	Non-vital	X	X	X	X	X	X	X	X
	LmandI1	Non-vital	X	X	X	X		X	X	X
4	RmaxM2	Non-vital	X				X		X	
6	LmandI3	Vital	X		X	X	X	X	X	X
7	RmaxP4	Vital	X		X	X	X	X	X	X
	RmaxM1	Non-vital	X	X	X		X	X		
8	RmaxC	Non-vital			X				X	
	LmaxC	Non-vital	X	X	X				X	
12	LmaxI2	Non-vital	X	X	X	X		X	X	X
14	LmaxP4	Non-vital	X	X	X	X			X	X
15	RmandI2	Non-vital	X	X				X		
	LmandI2	Non-vital	X				X			
	LmandI3	Non-vital	X				X		X	
	RmaxP4	Non-vital	X	X			X	X	X	

The table summarizes pulpal vitality as determined by histologic examination and the corresponding radiographic diagnoses obtained using cone-beam computed tomography (CBCT) and intraoral dental radiography (IDR). PAL, periapical lucency; FTN, failure to narrow; EIRR, external inflammatory root resorption; LCI, loss of crown integrity.

### Diagnostic performance relative to histopathology (*n* = 18)

Overall, both CBCT and IDR were highly sensitive for detecting non-vital pulps but showed limited ability to identify vital teeth.

**Figure 5:**
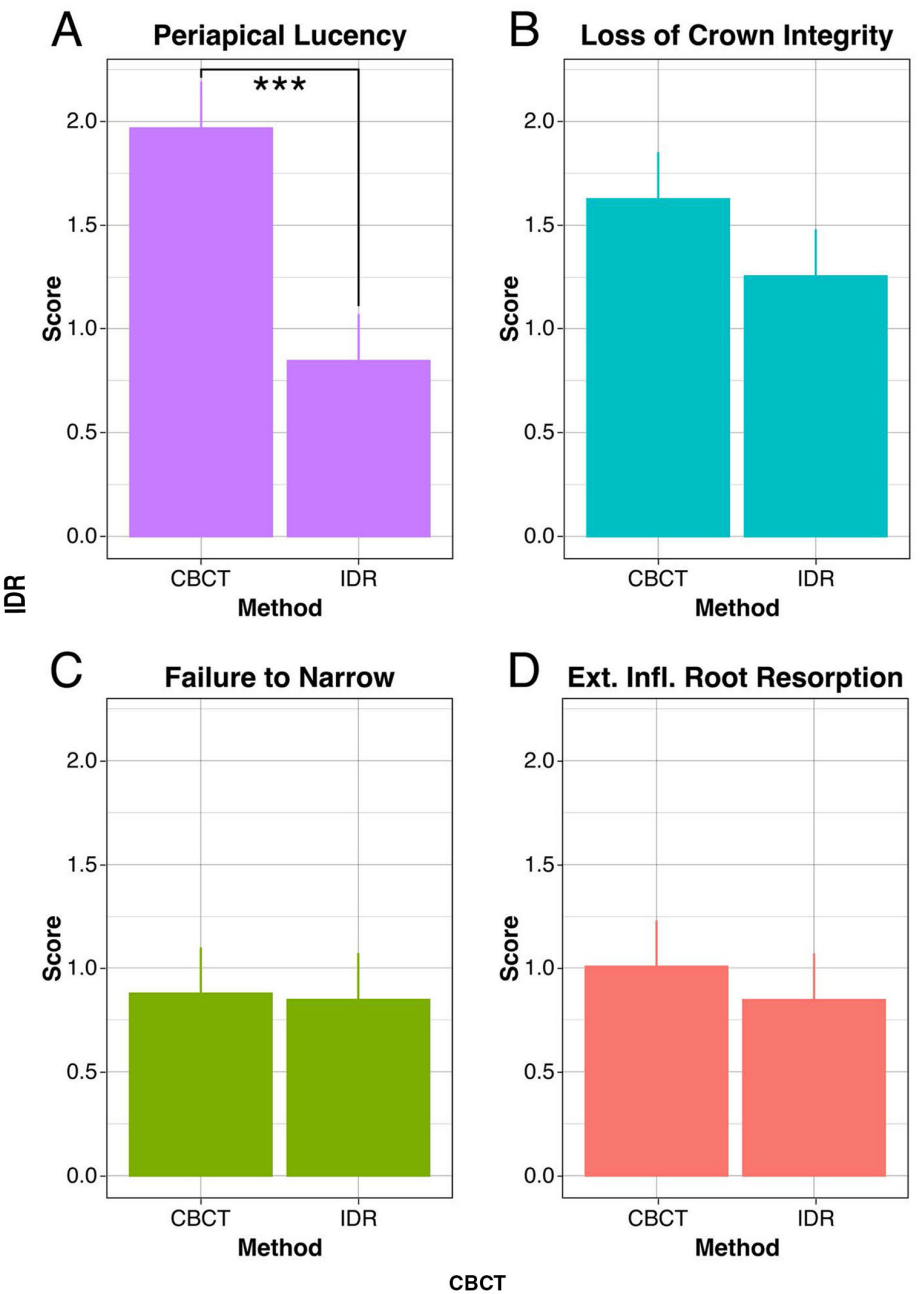
Mixed-effects model results comparing diagnostic scores between cone-beam computed tomography (CBCT) and intraoral dental radiography (IDR). CBCT yielded significantly higher diagnostic scores than IDR across raters and diagnostic category (*p* < 0.002), with a significant interaction between imaging modality and diagnostic category (*p* < 0.001). CBCT demonstrated markedly higher scores for periapical lucency (*p* < 0.001) the presence of the three asterisks indicates that the statistical significance refers to a *p* value < 0.001 **(A)**, whereas differences between modalities were marginal for loss of crown integrity **(B)** and not statistically detectable for failure of canal narrowing **(C)** or external inflammatory root resorption (EIRR) **(D)**.

Results were based on the aggregation method yielding the highest overall diagnostic performance. Using the best-performing method for combining rater scores, CBCT demonstrated higher sensitivity than IDR, but failed to correctly classify vital teeth (sensitivity = 0.88; specificity = 0; accuracy = 0.78). In contrast, IDR showed slightly lower sensitivity but higher specificity. While both modalities identified most non-vital teeth correctly, CBCT incorrectly classified all histologically vital teeth as non-vital in this dataset, whereas IDR correctly classified a subset of vital teeth (sensitivity = 0.88; specificity = 0.50; accuracy = 0.83) (see Figure 5).

### Effect of imaging modality and diagnostic features (*n* = 46)

Based on findings from mixed-effects models of rater score, CBCT consistently produced higher diagnostic scores than IDR across all teeth, indicating increased detection of imaging features associated with endodontal disease.

This difference was primarily driven by improved identification of periapical lucencies (PAL) with CBCT, whereas differences between modalities were minimal for other features, including loss of crown integrity, failure of canal narrowing, and external inflammatory root resorption (EIRR).

### Inter-rater agreement

Overall inter-rater agreement was moderate and varied according to diagnostic feature and imaging modality. Concordance analysis demonstrated that inter-rater agreement was generally limited by variability in the scores assigned to the same imaging findings rather than by systematic differences among evaluators. Accuracy coefficients exceeded 0.90 across most modality-feature combinations, indicating minimal systematic bias (Table 4).

**Table 4:** Inter-rater agreement according to imaging modality and diagnostic feature.

Diagnostic feature	Modality	Concordance correlation coefficient (CCC)	Accuracy coefficient	Precision coefficient
Periapical lucency (PAL)	CBCT	0.461	>0.90	0.509
Periapical lucency (PAL)	IDR	0.470	>0.90	0.470
Failure to pulp canal narrowing (FTN)	CBCT	0.675	>0.90	0.693
Failure to pulp canal narrowing (FTN)	IDR	0.574	>0.90	0.607
External inflammatory root resorption (EIRR)	CBCT	0.272	>0.90	0.293
External inflammatory root resoprtion (EIRR)	IDR	0.378	0.75	0.500
Loss of crown integrity (LCI)	CBCT	0.691	>0.90	0.700
Loss of crown integrity (LCI)	IDR	0.773	>0.90	0.789

Values represent the concordance correlation coefficient (CCC) and its accuracy and precision coefficients.

In particular, reduced agreement was observed for EIRR on IDR. Mixed-effects modeling identified rater-dependent differences in scoring, with two evaluators consistently assigning higher scores for EIRR than the remaining raters.

Additional analyses indicated that variability in scoring differed across imaging modalities and diagnostic features, but no consistent systematic bias between raters was identified.

## Discussion

This prospective study evaluated the diagnostic yield of cone-beam computed tomography (CBCT) compared with intraoral dental radiography (IDR) for the diagnosis of endodontal disease in dogs, using histopathology as the reference standard in a subset of teeth. Four principal findings emerged. First, CBCT demonstrated superior performance in the detection of periapical lucency, which represented the most robust and discriminative imaging feature across imaging modalities. Second, inter-rater agreement varied among diagnostic criteria and was particularly limited for external inflammatory root resorption (EIRR). Third, both CBCT and IDR exhibited high sensitivity and overall accuracy, but limited specificity and moderate inter-rater agreement. Finally, diagnostic performance was influenced by complex interactions among imaging modality, diagnostic criterion, rater, and patient, underscoring the multifactorial nature of endodontal disease image interpretation.

Among the four radiographic features evaluated, periapical lucency showed the clearest distinction between imaging modalities, with CBCT yielding significantly higher diagnostic scores than IDR across all raters. This finding supports the hypothesis that three-dimensional imaging enhances visualization of periapical bone changes by eliminating superimposition of adjacent anatomical structures and geometric distortion inherent to two-dimensional radiography (20–22). These limitations of two-dimensional imaging have long been recognized. Experimental work by Bender and Seltzer demonstrated that lesions confined to cancellous bone may remain radiographically undetectable unless bone loss extends to involve the cortical plate, highlighting a fundamental constraint of conventional radiography in early lesion detection (23, 24). This principle is further supported by classic endodontic literature, which emphasizes that both cortical bone density and anatomical superimposition can obscure early periapical pathology. These advantages are particularly relevant in dogs, in whom complex root morphology and thick cortical bone may further obscure early periapical pathology on conventional radiographs (3). These advantages are clinically relevant, as improved detection and characterization of periapical pathology can directly inform treatment planning, including decisions regarding tooth extraction or endodontic therapy, thereby affecting patient outcomes.

The superior performance of CBCT for detecting periapical lucency observed in this study is consistent with previous veterinary and human investigations. In both fields, conventional radiography has been shown to underestimate the presence and extent of apical periodontitis, with a substantial proportion of histologically confirmed lesions lacking radiographic evidence on IDR (4, 7, 9, 10). Human studies similarly report that up to one quarter of apical lesions may be missed on periapical radiographs, particularly during early stages of disease development (11). These observations are consistent with foundational experimental evidence indicating that the detectability of periapical lesions depends on their spatial relationship to cortical bone, with lesions located centrally within cancellous bone being less likely to be identified radiographically (23–25). Collectively, these findings highlight periapical lucency as the radiographic feature most likely to benefit from volumetric imaging and reinforce the clinical value of CBCT for early detection of apical pathology. The presence of a periapical lucency has been associated with lower success rates for endodontic therapy, suggesting that earlier detection of apical pathology through CBCT may facilitate treatment before substantial periapical bone loss occurs, potentially improving prognosis (26).

Inter-rater agreement represented a significant limitation across both imaging modalities and was particularly reduced for EIRR. Statistical analysis demonstrated that disagreement was driven primarily by low precision rather than systematic bias, with notable rater-specific divergence for EIRR, especially on IDR. Two raters consistently assigned higher resorption scores on radiographs, contributing disproportionately to reduced agreement. These findings align closely with recent veterinary literature (9, 27). Feigin et al. (9) reported only fair overall agreement among veterinary students, residents, and specialists interpreting dental radiographs, with particularly poor agreement for resorptive lesions, even among experienced clinicians. Similar challenges have been documented in human endodontics, where external root resorption and subtle periapical changes are recognized as among the most difficult entities to interpret reliably (28, 29). Earlier human studies further underscore the subjective nature of radiographic interpretation. Goldman et al. reported agreement in only approximately 50% of cases among evaluators assessing periapical pathosis, with intra-observer consistency ranging from 75 to 83% upon reevaluation, highlighting inherent variability even among trained observers (30). The present results suggest that EIRR remains a diagnostically challenging entity, even when advanced imaging modalities are used. While CBCT may improve lesion conspicuity, interpretive variability persists, emphasizing the need for standardized diagnostic criteria, targeted training, and potentially adjunctive tools to improve rater convergence.

Both CBCT and IDR showed high sensitivity and overall accuracy for detecting non-vital teeth when compared with histopathology. However, specificity and precision were limited, leading to only moderate balanced accuracy. This pattern reflects the predominance of non-vital pulps in our caseload and indicates a tendency of both imaging modalities to classify some histologically vital teeth as non-vital. Importantly, several of these teeth exhibited clinically relevant endodontal pathology, including pulp exposure and histologic evidence of pulpitis. Consequently, these findings may not represent overdiagnosis of endodontal disease, but rather discordance between imaging-based assessment of pulpal status and histologic classification based on pulp vitality.

From a clinical perspective, high sensitivity may be advantageous for minimizing false-negative results and ensuring timely intervention in dogs with endodontal disease. However, limited specificity raises concern for false-positive diagnoses, which may lead to unnecessary extractions or endodontic treatment. Similar trade-offs between sensitivity and specificity have been reported in human studies comparing CBCT with IDR (12–15).

In this study, indeed, three teeth classified as non-vital based on imaging findings were subsequently categorized as vital on histopathologic examination. Closer inspection, however, suggests that these three cases represent a spectrum of pulpal conditions rather than unequivocal diagnostic discordance. In two cases, clear signs of pulpitis were identified, a condition widely regarded as a transitional stage preceding pulpal necrosis (33). In the third case, definitive evidence of pulpal vitality was limited, as no clearly viable odontoblasts were identified and only vascular endothelial structures were observed, precluding full confirmation of functional pulp vitality (33).

Importantly, pulp vitality should not be interpreted as synonymous with the absence of clinically significant endodontal disease. Teeth with pulp exposure and histologic evidence of pulpitis may remain vital despite requiring clinical intervention. Furthermore, in cases of complicated crown fractures or other forms of pulp exposure, the presence of a vital pulp at the time of histologic evaluation does not necessarily indicate a healthy pulp or a favorable long-term prognosis, as progression to pulp necrosis will occur without treatment (33). Consequently, some imaging findings classified as false positives relative to pulp vitality may still have represented clinically relevant endodontal disease. The reduced specificity observed in both CBCT and IDR should therefore be interpreted within the context of the reference standard used in this study, which assessed pulp vitality rather than the broader presence or absence of endodontic pathology. These findings highlight the inherent challenges in dichotomizing pulpal status and may partially explain the reduced specificity observed in both imaging modalities.

While reduced specificity may theoretically increase the risk of overtreatment if imaging findings are interpreted in isolation, treatment decisions in clinical practice should not be based solely on imaging results. Rather, radiographic findings are integrated with clinical examination findings, tooth history, evidence of pulp exposure or crown integrity, and patient-specific factors. Furthermore, in the present study, two of the three histologically vital teeth demonstrated pulpitis, suggesting that some apparent false-positive imaging findings may still have identified clinically relevant pathology warranting treatment. Nevertheless, the potential for overtreatment remains an important consideration and underscores the need to interpret advanced imaging findings within the broader clinical context.

Importantly, in our dataset, there was minimal systematic bias among raters as seen with high accuracy component of the concordance correlation coefficient (CCC) preserved across categories. Based on these findings, within the context of this study, interpretive uncertainty—rather than directional bias—appears to represent the main limitation of imaging-based diagnosis. Collectively, our results indicate that improving diagnostic consistency may depend less on correcting systematic bias and more on standardizing interpretive criteria and enhancing observer training.

Further, our findings, specifically the mixed effect modeling, emphasized that diagnostic yield cannot be fully attributed to imaging technology alone, but reflects a complex interplay of biological variability and personal interpretation. Such interactions mirror findings from prior veterinary imaging studies demonstrating that diagnostic yield varies across anatomic regions, lesion types, and observer experience (16, 17, 20, 21, 31, 32). Recognition of these factors is essential when integrating CBCT into clinical decision-making and underscores the importance of contextual interpretation rather than reliance on a single diagnostic sign.

An additional consideration relates to the exclusion of certain radiographic parameters, such as periodontal ligament space widening and integrity, which are traditionally included in the assessment of endodontal and periodontal disease (23, 24).

Their exclusion was intended to maintain consistency with previously established veterinary endodontic criteria and to focus on radiographic findings more strongly associated with pulpal disease (8). Inclusion of these additional variables may have improved diagnostic specificity by providing supplementary information regarding periapical tissue health and potentially aiding distinction between clinically healthy and diseased teeth. However, widening of the periodontal ligament space and loss of lamina dura integrity are not specific to endodontal disease and may also occur secondary to periodontal, traumatic, or other inflammatory processes (34). Because these parameters were not systematically evaluated in the present study, their actual contribution to diagnostic performance cannot be determined. Future investigations incorporating these radiographic findings may help clarify whether they enhance discrimination between clinically relevant endodontal disease and normal pulpal status while maintaining acceptable sensitivity.

Despite the compelling data presented especially concerning CBCT superiority and its enhanced capability of identifying periapical lucencies, it is essential to consider the inherent limitations of this study. The relatively small number of teeth subjected to histopathological evaluation constrained statistical power and contributed to limited specificity estimates. Additionally, the predominance of non-vital teeth may have biased performance metrics toward high sensitivity. Finally, although a structured scoring system was employed, qualitative assessment remains inherently subjective, and inter-rater variability could not be fully mitigated.

An additional limitation is that histopathologic evaluation was available for only a subset of enrolled teeth. Although tissue loss and non-diagnostic samples occurred for a variety of procedural reasons, including pulp desiccation, extraction-related tissue damage, sectioning artifacts, and inadvertent disposal of extracted teeth, these exclusions may not have been entirely random. It is possible that the subset of teeth with diagnostic histopathology overrepresented more advanced stages of endodontal disease, resulting in a higher prevalence of non-vital pulps than would be expected in the broader study population. Consequently, estimates of diagnostic performance, particularly sensitivity, may have been inflated, whereas specificity estimates should be interpreted cautiously. Future studies with more complete histopathologic sampling will be necessary to better define the true diagnostic performance of both imaging modalities.

In conclusion, CBCT demonstrated superior detection of periapical lucency compared with IDR, representing the most reliable imaging feature of endodontal disease in dogs. However, significant inter-rater variability, particularly for EIRR, and limited diagnostic precision highlight persistent challenges in imaging-based diagnosis. While CBCT offers meaningful advantages for detection and anatomical assessment, its clinical value is maximized when interpreted within a comprehensive diagnostic framework that accounts for observer variability, lesion type, and patient-specific factors. Future studies with larger cohorts and standardized interpretive protocols are warranted to further refine the role of CBCT in veterinary endodontic diagnostics.

## Data Availability

The raw data supporting the conclusions of this article will be made available by the authors, without undue reservation.
